# The Comparative Photodegradation Activities of Pentachlorophenol (PCP) and Polychlorinated Biphenyls (PCBs) Using UV Alone and TiO_2_-Derived Photocatalysts in Methanol Soil Washing Solution

**DOI:** 10.1371/journal.pone.0108765

**Published:** 2014-09-25

**Authors:** Zeyu Zhou, Yaxin Zhang, Hongtao Wang, Tan Chen, Wenjing Lu

**Affiliations:** 1 Department of Environmental Science and Engineering, Tsinghua University, Beijing, P.R. China; 2 College of Environmental Science and Engineering, Hunan University, Hunan, P.R. China; The University of Iowa, United States of America

## Abstract

Photochemical treatment is increasingly being applied to remedy environmental problems. TiO_2_-derived catalysts are efficiently and widely used in photodegradation applications. The efficiency of various photochemical treatments, namely, the use of UV irradiation without catalyst or with TiO_2_/graphene-TiO_2_ photodegradation methods was determined by comparing the photodegadation of two main types of hydrophobic chlorinated aromatic pollutants, namely, pentachlorophenol (PCP) and polychlorinated biphenyls (PCBs). Results show that photodegradation in methanol solution under pure UV irradiation was more efficient than that with either one of the catalysts tested, contrary to previous results in which photodegradation rates were enhanced using TiO_2_-derived catalysts. The effects of various factors, such as UV light illumination, addition of methanol to the solution, catalyst dosage, and the pH of the reaction mixture, were examined. The degradation pathway was deduced. The photochemical treatment in methanol soil washing solution did not benefit from the use of the catalysts tested. Pure UV irradiation was sufficient for the dechlorination and degradation of the PCP and PCBs.

## Introduction

Hydrophobic chlorinated aromatic pollutants, such as pentachlorophenol (PCP) and polychlorinated biphenyls (PCBs), are among the most important environmental pollutants in the twentieth century. For several decades after their commercial production, these compounds had been widely used for numerous applications, such as in wood protection, pesticides, and dielectric fluids in capacitors and transformers, before their global biota accumulation and genotoxic activity were gradually noticed [1]. Given their potential health hazard for humans and wildlife, the Stockholm Convention in 2001 classified PCP and PCBs as Persistent Organic Pollutants. Despite a comprehensive production ban since that time, millions of tons of these compounds continue to circulate in the environment [2], [3]. Thus, the dechlorination and degradation of PCP and PCBs have emerged as major issues.

The cleanup of hydrophobic chlorinated aromatic pollutants is a challenging task. Various remediation technologies have been developed because of the extremely slow natural degradation of these compounds [4]. Some of these techniques are based on a “dig and dump approach,” such as landfilling or capping. This method immobilizes the contaminant to prevent it from entering the aqueous phase [5]. Other methods are based on a “dig and incinerate approach,” such as thermal treatment in which heat is used to remove or destroy the contaminants [6]. Among all the remediation technologies, soil washing combined with photochemical treatments has become increasingly advantageous because this method does not release toxic by-products into the environment and the cost is reasonable [7]. Soil washing is a well-developed technology that removes contaminants from polluted solid phase. The target contaminant can be extracted from the soil by a soil washing solvent, and the following photocatalytic degradation can be altered with different soil washing solvents [8], [9]. Among the various solvents used to extract the target contaminant from the soil matrix, alcohol, such as methanol and ethanol, has been successfully used to remove PCP and other contaminants [10], [11]. The solvent contains target contaminants after soil washing, which are usually treated under ultraviolet light photolysis, and the organic contaminants inside can be decomposed by photodegradation [12]. During UV irradiation, •OH can be formed from water molecules or other highly reactive solvents, initiating the decomposition of the pollutants [13].

To enhance the degradation rate, different types of photocatalysts such as TiO_2_ and ZnO have been investigated for photodegradation [14]–[17]. In these studies, the photocatalytic degradations of PCP have been enhanced by various catalysts compared with TiO_2_ or pure UV irradiation. Although remediation systems using a photocatalyst combined with UV irradiation have been successfully demonstrated to treat heterogeneous polluted soil or water [18], [19], whether the addition of photocatalyst provideds more efficient photodegradation in any condition than using pure UV irradiation has not been proved. Considering that TiO_2_ has been used extensively for water treatment and control of organic contaminants, the heterogeneous modifications of TiO_2_ have been evaluated [20]–[23]. To demonstrate the improvements of modified TiO_2_ catalysts, numerous studies have conducted photodegradation competitions between new catalysts and the original TiO_2_ catalyst. Degradations using pure UV irradiation under analogous conditions were rarely investigated [21], [23], [24].

The different methods for treating PCP and PCBs in methanol soil washing solvent under UV irradiation with or without a TiO_2_-derived catalyst were compared in this study. TiO_2_ coupled with graphene was used as modified TiO_2_ catalyst in this study. Graphene has high electrical conductivity and efficient electron storage and shuttling capabilities. In this study, the degradation rates under UV irradiation with and without a TiO_2_-derived catalyst are presented. Although the degradation rates using modified TiO_2_ catalysts were all remarkably higher compared with the original TiO_2_ catalyst, they were not as high as that when only UV irradiation was used. The main factors influencing the degradation that were taken into consideration included UV light illumination, addition of methanol to the solution, catalyst dosage, and the pH value of the solution. The beneficial effect of adding a catalyst for the contaminant photodegradation in methanol soil washing solvent was not confirmed.

## Materials and Methods

### Reagents and materials

Commercial P25 TiO_2_ (80% anatase, 20% rutile) was supplied by the Degussa (Germany). Hexane and methanol (HPLC grade) were purchased from Fisher Scientific (USA). Graphene oxide was purchased from XFNano (China). Water, which was used as a solvent, was obtained using a Milli-Q Water Purification System. Pentachlorophenol (PCP, purity >98.0%) and pentachlorophenol sodium salt (PCP-Na) were purchased from the Sigma-Aldrich (USA). PCBs were obtained directly from a waste transformer factory in China. Methyl Orange (MO, analytical grade) was purchased from Sino Chemical Reagent (China).

Graphene-TiO_2_ was prepared via a hydrothermal method. 25 mg of graphene oxide were dispersed in 50 mL water via sonication for 1 h to form a stable solution. Then, 1 g of TiO_2_ was added into the graphene oxide suspension, which was stirred to mix the solution thoroughly. The mixture was moved into a Teflon-lined autoclave and heated at 180°C for 6 h. The resulting grey slurry was filtered and dried prior to use [24], [25].

### Photoirradiation procedure

**Figure 1:**
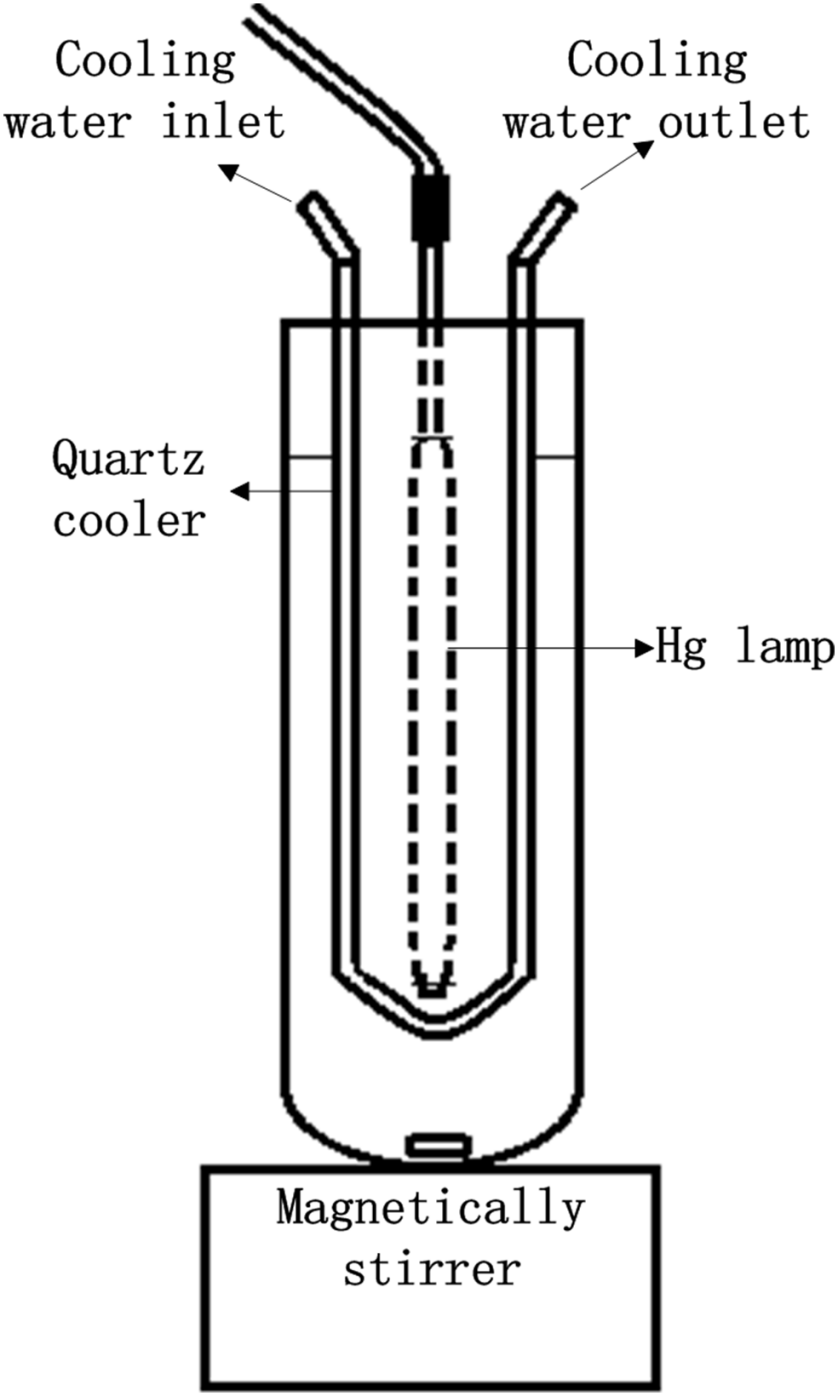
Schematic experimental system for photo degradation experiments.

All UV irradiation parts of the experiments were conducted in a 500 mL glass reactor (length 310 mm, diameter 70 mm), which contained a UV lighting system, as shown in Fig. 1. The lighting system included a high-pressure mercury lamp (GGZ300, Phillips, maximum wavelengths at 254, 292, 313, 334, 365, 436 and 546 nm) for 100 W and 300 W UV irradiation; a low-pressure mercury lamp (Hagende, maximum wavelength at 254 nm) for 9 W UV irradiation; a quartz well (length 300 mm, diameter 55 mm) equipped with a circulating water unit to maintain the system at 20°C; and a magnetic stirrer to promote uniform mixing of the catalyst in the solution. The UV light intensities at the reaction point were 2.0 * 10^3^ Lx for the 9 W mercury lamp, 7.2 * 10^4^ Lx for the 100 W mercury lamp, and approximately 3 * 10^5^ Lx for the 300 W mercury lamp, as measured by a Lux tester (Hioki, Japan). The radiation powers of UV light at the reaction point were 5.2 * 10^2^ µW cm^−2^ for the 9 W mercury lamp, 14.7 * 10^3^ µW cm^−2^ for the 100 W mercury lamp, and 72.3 * 10^3^ µW cm^−2^ for the 300 W mercury lamp, as measured by a radiometer (Handy, China).

Typically the reactor contained 500 mL of the soil washing solution with a concentration of 10 mg L^−1^ of target contaminant (e.g., 0.0375 mM PCP solution) with or without 200 mg catalyst prior to photodegradation [26]. After 2 h of equilibrating the solutions in the dark, UV light irradiation was initiated to start the reactions.

### Analytical methods

To follow the degradation process, 1 mL of mixture was extracted from the soil washing solution at the beginning, after equilibration, and after UV irradiation at predetermined time-points (5, 10, and 20 min for the 300, 100, and 9 W mercury lamps, respectively). Every mixture was adequately shaken with the same volume of methanol and then filtered through a 0.45 µm pore size membrane filter (for PCBs, every mixture was shaken with 2 mL of hexane).

PCP and phenol were analyzed using high-performance liquid chromatography (HPLC, Agilent 1260) equipped with an Agilent TC-C18 reverse phase column. UV detection was performed at 249, 270, and 254 nm for PCP, phenol, and other by-products, respectively [26]. A mixture of methanol and water was used as the mobile phase, with a gradient mixture and a flow rate of 1.0 mL min^−1^. The quantification of the target analytes was based on the calibration curve in the HPLC analysis, and the linear range was 0 mg L^−1^ to 10 mg L^−1^. Analytical results were verified by standard PCP and phenol solution with certain concentration.

PCBs were determined using a gas chromatography – mass spectrometry instrument (GC/MS-QP2010, Shimadzu, Japan) equipped with an HP-5MS capillary column. The temperature of the GC oven was held at 150°C for 1 min, increased to 185°C at a rate of 20°C min^−1^, followed by an increase to 245°C at a rate of 2°C min^−1^, and then held at 245°C for 3 min prior to further increase to 290°C at a rate of 6°C min^−1^. The injector and detector temperatures were 250 and 290°C, respectively. The carrier gas was helium, which was utilized at a flow rate of 1.0 mL min^−1^. The MS ion source and interface temperatures were 200 and 220°C, respectively.

The identification results were confirmed by GC/MS with the same HP-5MS capillary column and temperature control procedure as the GC analysis. Quantification of different PCB congeners was caculated based on the peak areas of their respective response factors of an authentic standard [27].

## Results and Discussion

### Photodegradation of PCP under UV irradiation

**Figure 2:**
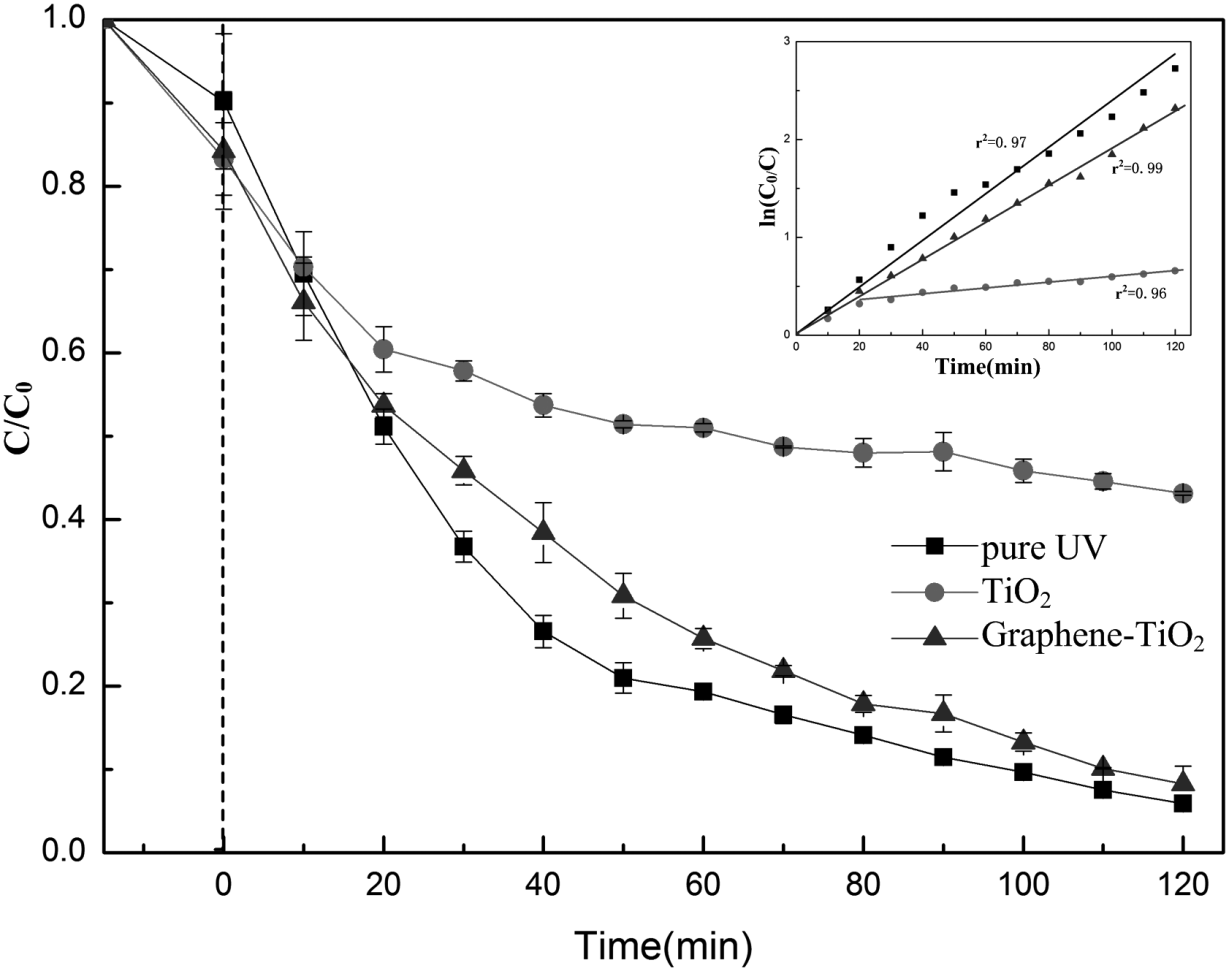
Photocatalytic degradation of PCP with TiO_2_, graphene-TiO_2_ and without catalyst under 100 W UV light. The inset represents the logarithmic transform for each curve.

To explore the photodegradation rate under UV illumination, variations in the concentrations of PCP were investigated. The PCP photoactivities under 100 W UV light with TiO_2_, with graphene-TiO_2_, and without catalyst are compared in Fig. 2. The initial PCP concentration in the solution was 10 mg L^−1^ (0.0375 mM) and the solvent was a 1∶100 methanol–water soil washing solution. The photocatalytic activity using TiO_2_ was considerably lower than those in the other two treatments, and pure UV (without a catalyst) showed the highest activity. After 120 min of UV irradiation, the removal rates of PCP by pure UV (without a catalyst), UV with graphene-TiO_2_, and UV with TiO_2_ were 94%, 92%, and 57%, respectively.

The logarithm of the ratio between the initial concentration (C_0_) of PCP and its concentration (C) at a specific given time is shown in the inset of Fig. 2. The slope of these straight lines provided the apparent rate constant. All correlation coefficients (r^2^) obtained were higher than 0.95. As shown in this figure, pure UV irradiation of the PCP solution provided the highest photocatalytic rate constant, namely, 0.0236 min^−1^. The photodegradation rate constant using graphene-TiO_2_ was slightly lower at 0.0191 min^−1^. The data for the photodegradation using TiO_2_ was not linear at the beginning of the irradiation procedure. Thus, the slope was measured using data corresponding to 20 min or more of photoirradiation, which provided a photocatalytic rate constant of 0.0031 min^−1^. This value was almost 1/8 of that of the highest measured rate constant. The result showed that the PCP can react with the •OH in a solution formed by UV irradiation and undergo degradation. The higher photodegradation rate using pure UV light than those with catalyst could be attributed to two main reasons. First, the C–O bond energy of methanol compound was lower than the C–Cl bond energy of the PCP compound, and the methanol compound is much easier attached to the surface of TiO_2_ than hydrophobic chlorinated aromatic pollutant. When photo irradiation began, the methanol attached to the TiO_2_ surface was degraded first. Second, the UV light, which could act on PCP was blocked and absorbed by TiO_2_ catalyst and led to an energy loss. The high photocatalytic rate constant at the beginning of the photodegradation procedure was also caused by two reasons. First, soil washing solutions were equilibrated with the photocatalyst prior to irradiation. The high concentration of the target contaminant resulted in their adsorption on the catalyst surface, which reacted easily with the photoexcited electrons when UV irradiation began. Thus, the energy wasted was minimal. After a period of reaction, the pollution on the catalyst surface was degraded, and the photodegradation rate decreased. Second, when mercury lamp was turned on briefly, the energy in UV region was slightly higher, which also led a higher constant rate at the beginning.

### Photodegradation of PCBs under UV irradiation

**Figure 3:**
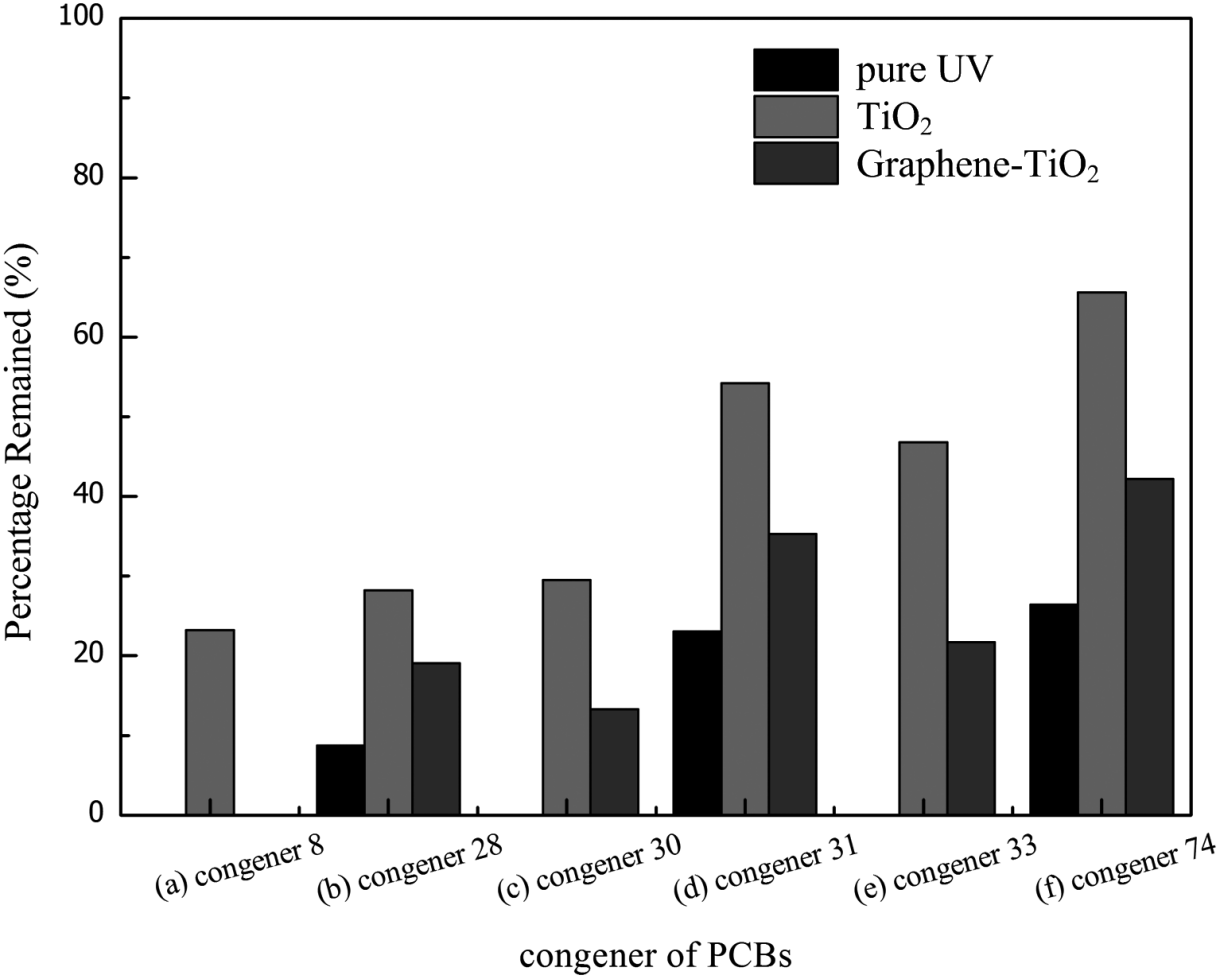
Percentage remained of PCBs after 2 hours photodegradation under UV light with TiO_2_, graphene-TiO_2_ and without catalyst: (a) congener 8; (b) congener 28; (c) congener 30; (d) congener 31; (e) congener 33; (f) congener 74.

The experiments with the mixture of PCBs were complex because various PCB congeners were present in the sample, and excessive by-products were created during photodegradation. The experiments were simplified by monitoring the concentrations of six major components to explore the photodegradation rate of PCBs in methanol soil washing solution under 100 W UV irradiation. The initial concentration of PCBs in the solution was 10 mg L^−1^, and the solvent was a 1∶10 methanol–water soil washing solution. The concentrations of the six major components in the solution were 0.67 mg L^−1^ 2,4′-dichlorobiphenyl (congener 8), 1.48 mg L^−1^ 2,4,4′-trichlorobiphenyl (congener 28), 0.61 mg L^−1^ 2,4,6-trichlorobiphenyl (congener 30), 0.83 mg L^−1^ 2,4′,5-trichlorobiphenyl (congener 31), 0.68 mg L^−1^ 2′,3,4-trichlorobiphenyl (congener 33), and 0.58 mg L^−1^ 2,4,4′,5-tetrachlorobiphenyl (congener 74). Figure 3 compares the percentage of photodegradation for each PCB congener under UV irradiation either with TiO_2_, with graphene-TiO_2_ or without catalyst.

The results in Fig. 3 indicated that all congeners obtained the highest photodegradation rates using pure UV irradiation. The half-life of congener 8 was the shortest among the six congeners. Congener 8 in the mixture of PCBs was fully photodegraded in 20 min using pure UV irradiation but required 100 min if graphene-TiO_2_ was present in the mixture. For reactions that contained TiO_2_, congener 8 was degraded by 77% after 2 h of reaction time. Congener 30 was completely removed after 110 min using pure UV irradiation. The degradation rates were 71% and 87% after 2 h of reaction for systems with extra TiO_2_ and graphene-TiO_2_, respectively. Congener 33 was eliminated after 100 min, which was slightly faster than congener 30. However, the degradation rates of congener 30 using TiO_2_ and graphene-TiO_2_ as the catalysts were 53% and 78% after the reaction, respectively. These values were lower than those observed for congener 30. Congeners 28, 31, and 74 remained in each of the mixtures after the photodegradation, and their degradation rates under these experimental conditions were similar. The degradation rates were 91%, 72%, and 81% for congener 28, 77%, 46%, and 65% for congener 31, and 74%, 34%, and 58% for congener 74. The photodegradation of PCBs did not quite follow the pseudo-first order kinetics as described in previous articles [9], [12]. This finding may be caused by the fact that some PCB congeners may be parts of the pathway of another reagent, such as congeners 28 and congener 31, which can be formed from congener 74 via the dechlorination of one chlorine atom. The concentration of biphenyl could not be tested for the entire duration of all three procedures because its degradation rate was higher than those of the PCBs [9]. Chlorine atoms in the PCB molecules break apart from biphenyl during UV irradiation. When the number of the chlorine atoms in the PCB congeners decreases, they separate easily. PCB molecules will be dechlorinated gradually to biphenyl and then be decomposed to small molecule. Similar to PCP degradation, the photocatalytic activity of TiO_2_-derived catalysts was based on creating electron-hole pairs when exposed to UV radiation. The photoexcited electrons and holes were mainly reacting with the methanol in the solution, and the decomposition of target pollutants was delayed. The electron-hole pairs created on the photocatalyst surface were barely acting on the PCBs, and the block of UV light caused an energy loss, especially for the original TiO_2_ catalyst.

### Photodegradation of Methyl orange under UV irradiation

To certify that TiO_2_ could increase the photodegradation rate in other conditions under UV irradiation, Methyl orange (MO), a commonly used dye, was photodegraded. This dye could be easily trapped by the holes on the catalyst surface [28]. The reactor contained 500 mL of 50 mg/L MO (0.15 mM) with/without 1 g L^−1^ of TiO_2_ before photo degradation. This system was illuminated with 300 W UV lamp after adsorption-desorption equilibrium. To evaluate the discoloration rate of MO, the mixture withdrawn from the MO solution at every predetermined time-point was filtered through 0.45 µm pore size membrane filter and analyzed by UV-Vis spectroscopy at 463 nm (UV-2401PC, Shimadzu, Japan).

The degradation of MO solution fitted the pseudo first-order kinetic as reported. The significant degradation rates between pure UV light and UV with TiO_2_ were consistent with previously reported results [28]. Photo degradation using TiO_2_ as catalyst obtained a photocatalytic rate constant of 0.0759 min^−1^, which was significantly higher than that of pure UV irradiation which was 0.0010 min^−1^. After 60 min UV irradiation, the removal efficiency of MO using TiO_2_ was 99%, whereas that of MO using pure UV was only 6%. This result proved that TiO_2_ is actually useful in another condition.

### Effect of different UV illumination sources

**Figure 4:**
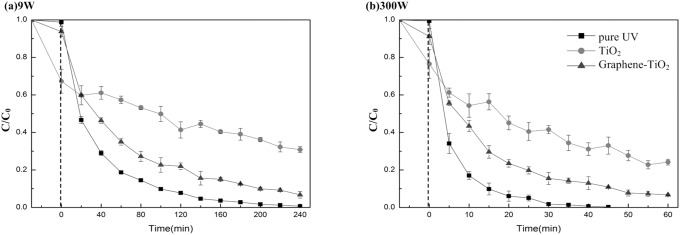
Photocatalytic degradation of PCP with TiO_2_, graphene-TiO_2_ and without catalyst under: (a) 9 W UV light; (b) 300 W UV light.

To investigate the relationship between the photodegradation rate and the UV illumination type, two groups of experiments were conducted to degrade PCP using mercury lamps of different intensities, as shown in Fig. 4. The same 300 W high-pressure mercury lamp was used. The power was increased from 100 W to 300 W. The results were compared with those obtained using a 9 W low-pressure mercury lamp. The apparent photocatalytic rate constant for each UV irradiation procedure is listed in Table 1. Pure UV photodegradation resulted in the highest photocatalytic rate constant at each of the different mercury lamp power intensities. Systems with TiO_2_ as a catalyst resulted in the lowest rate constants. The photocatalytic rate constants measured using the 9 W UV lamp were almost similar to those using the 100 W UV lamp. This result was ascribed to the fact that the low-pressure mercury lamp offered a maximum wavelength at 254 nm, which concentrated all the energy in the UV region. By contrast, the energy from the high-pressure mercury lamp was separated both in the UV and the visible areas. In other words, the energy from a low-pressure mercury lamp (in this case, the 9 W UV lamp) was fully utilized in the photodegradation procedure.

**Table 1:** Comparation of photocatalytic rate constants (k) of PCP with different photocatalyst conditions under 3 kinds of UV irradiation.

Photocatalyst	k (min^−1^,100 W UV)	k (min^−1^,300 W UV)	k (min^−1^,9 W UV)
Pure UV	0.0236	0.1317	0.0208
TiO_2_	0.0031	0.0211	0.0032
Graphene-TiO_2_	0.0191	0.0493	0.0116

### Effect of adding methanol and the mineralization

Methanol – water mixture was used as soil washing solvent to extract PCP or PCBs from contaminated soil for former photodegradation experiments. To estimate the influence of methanol and evaluate the mineralization of the pollution, PCP-Na was used for UV irradiation because it is soluble in water. A solution that contained 10 mg L^−1^ PCP-Na (0.0347 mM) was degraded without additional methanol using a 100 W UV light source. Following the aforementioned method for irradiation and data analysis, a photocatalytic rate constant of 0.0429 min^−1^ was measured, which was higher than that (0.0236 min^−1^) when 10 g L^−1^ methanol was included in the solution. The photodegradation rate of 10 mg L^−1^ PCP in pure methanol soil washing solution was also tested. After the same photo irradiation method, the photocatalytic rate constant was measured at 0.0143 min^−1^. This result indicated that methanol does not participate in the degradation pathway of PCP. The reaction of methanol consumes energy from the UV irradiation, which reduces the photocatalytic rate constant for target contaminant. The total organic carbon (TOC) of the solution was tested. TOC was 2.0 mg L^−1^ at the beginning while there is only PCP-Na in the solution. TOC decreased to approximately 80% when the PCP-Na in the solution was almost fully degraded, which indicated that dechlorination, degradation and mineralization were coexistent. After 2 h of UV irradiation, PCP-Na was fully removed and TOC decreased a half to 1.0 mg L^−1^, which showed that mineralization go along after PCP degraded.

### Phenol production after UV irradiation of PCP

The main contaminant in the solution was PCP during the UV irradiation procedure, because the photodegradation rate of PCP was lower than those of other types of chlorophenols on its degradation pathway [29], [30]. Chlorophenols produced in the degradation pathway of PCP were dechlorinated or decomposed because of their comparatively high photodegradation rate constant, and their concentration was undetectable by HPLC in the overall mixture. During UV irradiation, PCP lost chlorine atoms one by one until all Cl atoms were separated from the benzene ring at the beginning of the process. The dechlorination product of chlorophenols was phenol before further conversion, which resulted in the splitting of the benzene ring. The final concentration of phenol was investigated after each UV irradiation, as shown in Table 2. The results indicated that the final concentration of phenol in solution were similar for the three treatments. Given that the concentrations of other chlorophenols were negligible in the solution, PCP degradation did not end after dechlorination, whereas benzene ring continued to split. The photodegradation with pure UV irradiation was similar to the treatments using a catalyst, which did not simply terminate after dechlorination.

**Table 2:** Comparation of phenol production under different photocatalyst conditions after 2 hours UV irradiation and its percentage of initial PCP.

Photocatalyst	Concentration (mM)	Percentage phenolproduction of initial PCP
No catalyst	0.0057	15%
TiO_2_	0.0067	18%
Graphene-TiO_2_	0.0050	13%

### Effect of catalyst dosage

**Figure 5:**
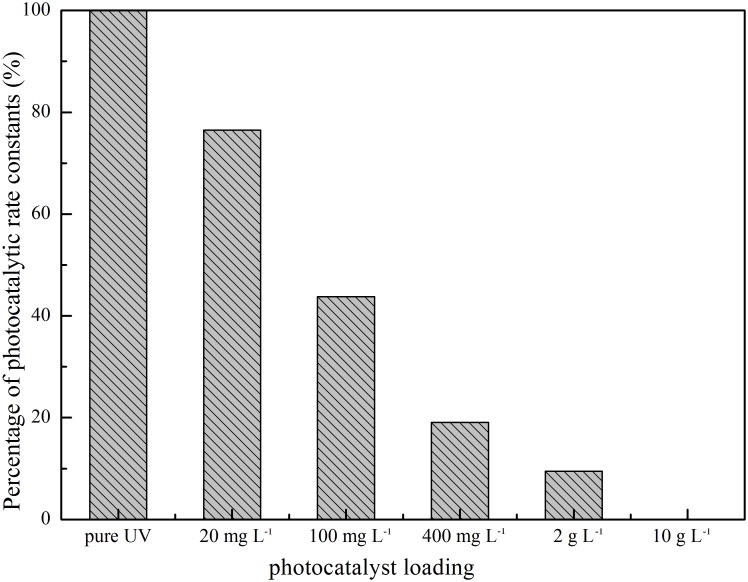
Photocatalytic rate constants of PCP degradation with different TiO_2_ loading, compared with the one of pure UV irradiation.

The effect of photocatalyst loading was investigated to determine the relationship between the absorption of photons and UV light energy blocked by the excess amount of catalyst. Therefore, a series of experiments with different amounts of TiO_2_ was conducted. The same 1∶100 methanol – water soil washing solution that contained 10 mg L^−1^ PCP (0.0375 mM) was degraded using a 100 W UV light source. All experiments followed pseudo-first order kinetics. The photocatalytic rate constant of each experiment was compared with that of pure UV irradiation, as shown in Fig. 5. Photodegradation rate decreased with increasing TiO_2_ loading. TiO_2_ catalyst almost blocked the whole photon energy when loading was up to 10 g L^−1^. The result indicated that the reaction with •OH in methanol soil washing solution is the main source of PCP degradation under UV light, and TiO_2_ added reduced the degradation.

### Effect of pH

**Figure 6:**
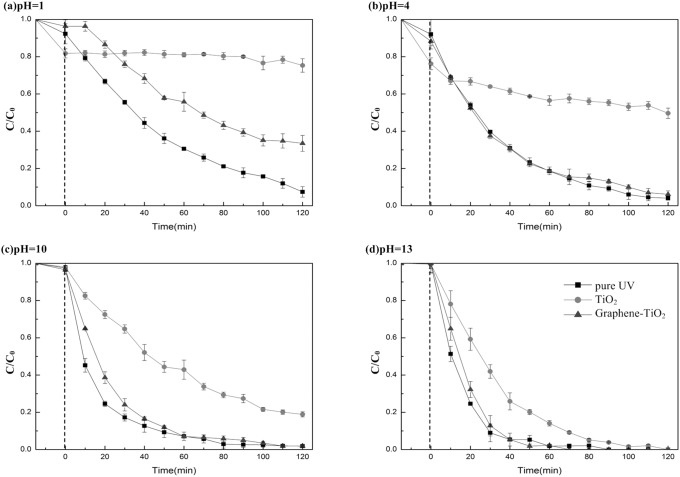
Photocatalytic degradation of PCP with TiO_2_, graphene-TiO_2_ and without catalyst under different pH values: (a) pH = 1; (b) pH = 4; (c) pH = 10; (d) pH = 13.

The pH values of the soil washing solution remained at approximately 4. To investigate the effect of pH on the photodegradation rate, PCP solutions with four different pH levels ranging from pH 1 to 13 were tested in the photoirradiation experiments using a 100 W UV light source, as shown in Fig. 6. The pH values were adjusted using either HCl or NaOH. The photodegradation rate increased as the pH value increased in each of the solutions. At high initial pH, the elevated concentration of the hydroxide ions (OH^−^) is assumed to result in increased •OH production that could accelerate the photodegradation rate. The pure UV irradiation and graphene-TiO_2_ catalyzed systems provided significantly higher degradation rates compared with those measured using TiO_2_ at each of the different pH values. The degradation rate of PCP under pure UV irradiation was slightly higher than that of graphene-TiO_2_ catalyzed systems at not so high pH level, whereas their rates became almost similar under alkaline conditions. When pH was too low, •OH was difficult to form, especially when considerable photon energy was blocked by TiO_2_. Thus, the photocatalytic activity of TiO_2_ was lost when the pH of the solution was adjusted to 1 using HCl.

## Conclusions

TiO_2_-derived catalysts are increasingly being used to treat samples that contain environmental pollutants, However, they are not well suited for the photodegradation of PCP or PCBs in methanol soil washing solution. Using graphene-TiO_2_ as a photocatalyst evidently enhanced the photodegradation rate under UV light irradiation compared with using TiO_2_ as the photocatalyst. However, pure UV irradiation showed the highest photocatalytic rate among the three treatment conditions. PCP and PCBs exhibited photoreactivities via •OH in the solution and were decomposed directly under UV irradiation. Addition of TiO_2_-derived catalysts led to a loss of energy in this condition, thereby decelerating photodegradation. The photodegradation abilities were similar under UV irradiation regardless of whether a photocatalyst was added because all treatments completed the dechlorination and degradation, which were similar to published results [9], [12]. The fact that biphenyl in the PCB solutions was undetectable during the entire reaction progress and the TOC test of PCP also illustrates that photodegradation under UV irradiation without a catalyst allows both dechlorination and degradation for the contaminants. Prior degradation of methanol and UV light blocked by the photocatalyst suspension caused photoenergy loss and a decrease in the photodegradation rate.
